# Effect of subcutaneous tocilizumab treatment on work/housework status in biologic-naïve rheumatoid arthritis patients using inverse probability of treatment weighting: FIRST ACT-SC study

**DOI:** 10.1186/s13075-018-1647-3

**Published:** 2018-07-20

**Authors:** Yoshiya Tanaka, Hideto Kameda, Kazuyoshi Saito, Yuko Kaneko, Eiichi Tanaka, Shinsuke Yasuda, Naoto Tamura, Keishi Fujio, Takao Fujii, Toshihisa Kojima, Tatsuhiko Anzai, Chikuma Hamada, Yoshihisa Fujino, Shinya Matsuda, Hitoshi Kohsaka

**Affiliations:** 1 University of Occupational and Environmental Health, 1-1 Iseigaoka, Yahatanishi-ku, Kitakyushu, Fukuoka, 807-0804 Japan; 2 Toho University, 2-22-36 Ohashi, Meguro-ku, Tokyo, 153-8515 Japan; 3 Tobata General Hospital, 1-3-33 Fukuryugi, Tobata-ku, Kitakyushu, Fukuoka, 804-0025 Japan; 4 Keio University School of Medicine, 35 Shinanomachi, Shinjuku-ku, Tokyo, 160-8582 Japan; 5 Tokyo Women’s Medical University, 10-22 Kawada-cho, Shinjuku-ku, Tokyo, 162-0054 Japan; 6 Hokkaido University, N15, W7, Kita-ku, Sapporo, Hokkaido 060-8638 Japan; 7 Juntendo University School of Medicine, 2-1-1 Hongo, Bunkyo-ku, Tokyo, 113-8421 Japan; 8 The University of Tokyo, 7-3-1 Hongo, Bunkyo-ku, Tokyo, 113-8654 Japan; 9 Wakayama Medical University, 811-1 Kimiidera, Wakayama, 641-8509 Japan; 10 Nagoya University Graduate School of Medicine, 65 Tsurumai-cho, Showa-ku, Nagoya, Aichi 466-8550 Japan; 11 EPS Corporation, 6-29 Shinogawamachi, Shinjuku-ku, Tokyo, 162-0814 Japan; 12 Tokyo University of Science, 6-3-1 Niijuku, Katsuhika-ku, Tokyo, 125-8585 Japan; 13 Tokyo Medical and Dental University, 1-5-45 Yushima, Bunkyo-ku, Tokyo, 113-8510 Japan

**Keywords:** Disease activity, Work disability, Quality of life, Rheumatoid arthritis, Tocilizumab

## Abstract

**Background:**

Following the onset of rheumatoid arthritis (RA), patients experience a functional decline caused by various joint symptoms which affects their activities of daily living and can lead to reduced work productivity. We evaluated the effect of a 52-week treatment with tocilizumab by subcutaneous injection (TCZ-SC) among biologic-naive Japanese house workers (HWs) and paid workers (PWs) with RA in a real-world clinical practice.

**Methods:**

This multicenter, observational, prospective study enrolled 377 and 347 RA patients into TCZ-SC and conventional synthetic disease-modifying antirheumatic drugs (csDMARDs)-alone groups, respectively. The primary endpoint was the change in percentage of overall work impairment (OWI) among PWs at week 52 assessed using the Work Productivity and Activity Impairment Questionnaire (WPAI). Inverse probability of treatment weighting analyses were used to compare treatments. The Work Functioning Impairment Scale, disease activity, quality of life (QOL) measures, and safety were also assessed.

**Results:**

The weighted change in OWI from baseline for PWs was −18.9% (TCZ-SC group) and −19.0% (csDMARDs group) at week 52, without a significant between-group difference (adjusted treatment difference 0.1, 95% confidence interval (CI) −6.3 to 6.5; *P* = 0.978). Changes in WPAI activity impairment in the overall group (between-group difference −6.4, 95% CI −10.7 to −2.2; *P* = 0.003) and HWs (−9.5, 95% CI − 16.0 to −2.9; *P* = 0.005) were significantly better with TCZ-SC than with csDMARDs at week 52. TCZ-SC-treated HWs showed significant improvement in all QOL assessments (Frenchay Activities Index, EuroQol 5 Dimension (EQ-5D), Japanese Health Assessment Questionnaire Disability Index (HAQ-DI), and 6-item Kessler scale (K6)) at week 52; PWs did not show any between-group differences for these QOL measures. Disease activity (Disease Activity Score 28-erythrocyte sedimentation rate, Clinical Disease Activity Index, and Simplified Disease Activity Index) and QOL measures (EQ-5D, HAQ-DI, and K6) improved over time in the overall group. No new safety concerns were raised with TCZ-SC.

**Conclusions:**

Despite the lack of differences in OWI between groups at week 52, the overall group (particularly HWs) receiving TCZ-SC in addition to csDMARDs showed significant improvements in activity impairment, disease activity, and QOL versus those receiving csDMARDs alone. This study may promote the evaluation of work productivity improvements in HWs and PWs by RA treatment.

**Electronic supplementary material:**

The online version of this article (10.1186/s13075-018-1647-3) contains supplementary material, which is available to authorized users.

## Background

The decrease in work productivity caused by rheumatoid arthritis (RA), whether it be for paid work or housework, has been gaining increasing attention [1–3]. Participation in work-related activities was added as one of the overarching principles of the primary goals of RA treatment [4]. It is estimated that, 6 months after the onset of RA, patients experience functional decline secondary to joint symptoms caused by joint inflammation and cartilage destruction. This not only affects activities of daily living, such as home activities, recreation, and social relations, but also results in reduced work productivity among house workers (HWs) and paid workers (PWs) [5–10]. Furthermore, it has been reported that, in Japanese patients with RA, work productivity and activity impairment are strongly correlated with the extent of physical disability and quality of life (QOL) [11].

The assessment methods for work productivity status are absenteeism (the decrease in number of actual working days by disease), presenteeism (the loss in the demonstration of the subject’s original working ability by disease activity), and overall work impairment (OWI; the sum of absenteeism and presenteeism). The Work Productivity and Activity Impairment Questionnaire (WPAI) is one of the recommended assessment methods for work productivity in RA patients [12, 13].

Recent advancements in the understanding of the molecular and cellular mechanisms of RA have led to the identification of novel targets and the development of effective biologic agents, such as tumor necrosis factor inhibitors [14] and anti-interleukin (IL)-6 receptor antibodies [15]. Tocilizumab (TCZ) is an anti-IL-6 receptor antibody that blocks the IL-6 receptor and inhibits the binding between IL-6 and its receptor. TCZ (in solution for intravenous administration) was approved for the treatment of RA in Japan in April 2008, in Europe in 2009, and in the US in 2010. Additionally, TCZ by subcutaneous injection (TCZ-SC) was approved in Japan in March 2013; thus, there are now two formulations available for RA patients.

No clinical studies have reported on the efficacy of TCZ in improving work productivity, either for paid work or housework, among RA patients. Therefore, in this study, we evaluated the effect of TCZ-SC based on improvements in work productivity and activity impairment among biologic-naive Japanese HWs and PWs with RA in a real-world clinical practice.

## Methods

### Study design

This was a multicenter, observational, prospective study in which patients were enrolled by central registration from 82 participating centers in Japan. The planned study period spanned from October 2013 to September 2015. The planned observation period was from October 2013 to December 2017.

The treatment period was 104 weeks for the TCZ-SC ± conventional synthetic disease-modifying antirheumatic drugs (csDMARDs) group, and 52 weeks for the csDMARD-alone group. As the main report of this research, we focus on reporting the comparison between treatment groups at 52 weeks.

### Patients

The inclusion criteria were: diagnosis of RA according to the 2010 American College of Rheumatology (ACR)/European League Against Rheumatism (EULAR) 2010 Classification Criteria; previous treatment with more than one csDMARD; performing remunerated work as an employee of a given company or family business (i.e., PW), or performing a central role in the housework within a household (i.e., HW); Disease Activity Score in 28 joints using the erythrocyte sedimentation rate (DAS28-ESR) ≥ 3.2; biologic-naive; prescribed TCZ-SC for the first time; receiving a csDMARD (except for tofacitinib) dose increase; receiving a csDMARD (except for tofacitinib) as add-on therapy; switching to a csDMARD (except for tofacitinib) treatment from other csDMARD(s); and written informed consent. Patients with any contraindication for use of the drugs evaluated in this study and those judged as ineligible for participation in this study by the investigators were excluded.

### Study oversight and conduct

Ethical approval was obtained from the institutional review board of each institution. The study was conducted in accordance with the Declaration of Helsinki and “Ethical Guidelines for Clinical Research” of the Ministry of Health, Labour and Welfare. Urgent events, such as adverse events (AEs), were reported to the research steering committee. Accordingly, the institutional review board and research steering committee determined the continuity of the patients in the study as well as that of the study itself. All patients provided written informed consent to participate in this study before being registered in the electronic data capturing system.

### Study treatment

In the TCZ-SC ± csDMARDs group, the dose was prescribed by the treating physician according to the prescribing information in the package insert [16]. In the csDMARDs-alone group, the dose of each csDMARD was prescribed according to the prescribing information in the corresponding package insert. Starting a csDMARD alone or in combination, as well as dose changes, switching to other csDMARDs, or adding other csDMARDs was permitted in the TCZ-SC ± csDMARDs group. Dose changes, switching, or addition of another csDMARD was also permitted in the csDMARDs-alone group.

### Assessments

The registration questionnaire was obtained at study registration. Patient demographic and disease characteristics were evaluated at baseline. The WPAI, Work Functioning Impairment Scale (WFun) [17], Frenchay Activities Index (FAI) [18], EuroQol 5 Dimension (EQ-5D) [19], Japanese Health Assessment Questionnaire Disability Index (HAQ-DI) [20, 21], and 6-item Kessler psychological distress scale (K6) [22] were assessed at baseline and at weeks 12, 24, and 52. DAS28-ESR, Clinical Disease Activity Index (CDAI) [23], and the Simplified Disease Activity Index (SDAI) [23] were assessed at baseline and at weeks 12, 24, 36, and 52. AEs were assessed continuously. The duration of assessments was approximately 52 weeks plus an additional 28 days (allowance).

Discontinuation criteria were as follows: 1) patient withdrawal; 2) physician’s decision because of AEs; 3) patients in the TCZ-SC ± csDMARDs group who switched from TCZ-SC to other biological agents; 4) patients in the csDMARDs-alone group who started treatment with biological agents, including TCZ and/or tofacitinib; and 5) other cases judged to require discontinuation by treating physicians.

### Endpoints

The primary endpoint was the change in the percentage of OWI among PWs at week 52 as assessed using the WPAI. The secondary endpoints for efficacy were as follows: change in the percentage of presenteeism (in PWs), absenteeism (in PWs), and activity impairment of daily work by WPAI (PWs and HWs); change in employment rate by WPAI (PWs); changes in WFun (PWs); and changes in disease activity by DAS28-ESR, CDAI, and remission rate. WPAI parameters were scored in the following way: absenteeism = (hours absent from work due to RA) / (hours absent from work due to RA + hours actually worked); and percentage of OWI = absenteeism + [(1 − absenteeism) × presenteeism].

The secondary endpoints for QOL were as follows: changes in FAI among HWs; changes in EQ-5D; changes in HAQ-DI (some questions were replaced to accommodate Japanese lifestyle differences and have been validated/confirmed) [20]; and changes in K6 improvement factor.

Additional exploratory analyses were conducted to assess the relationship between characteristics and each assessment outcome. Safety was assessed based on all AEs reported.

### Sample size calculation

Based on previous studies in Japan and the US reporting WPAI of PWs with RA as the main endpoint [5, 24], we assumed that the mean percentage of OWI (primary endpoint) was 30% to 40% at baseline. We also assumed that the percent change in OWI from baseline in the TCZ-SC and csDMARDs-alone groups at week 52 would be 40% and 15% (i.e., − 12% and −4.5% change from baseline considering a value of 30% at baseline), respectively. We used the Monte Carlo Simulation, repeated 10,000 times, to investigate the target population. Using the Wilcoxon rank sum test, we calculated the sample size to achieve a 5% two-sided significance level of 5% and 80% power. As a result, we estimated the need for a total of 160 PWs in both groups. Considering a possible drop out/discontinuation rate of 50% among PWs, we set the target population at 800 patients: 400 patients in the TCZ-SC ± csDMARDs group and 400 patients in the csDMARDs-alone group. Patient enrollment was continued until the number of PWs (excluding HWs) reached at least 200 in the TCZ-SC ± csDMARDs group.

### Study population

Efficacy analysis sets were the intention-to-treat set (patients whose treatment plan was determined among registered patients, except for any patient who did not provide written informed consent, or duplicated patients) and the modified intention-to-treat (mITT) set (patients in the TCZ-SC or csDMARDs-alone groups who received TCZ-SC or corresponding csDMARDs one or more times, except for patients with significant protocol deviations such as erroneous registration, lacking data for efficacy evaluations, or lacking baseline data for propensity score estimation). The safety analysis set included all patients in the TCZ-SC or csDMARDs-alone groups who received TCZ-SC or the corresponding csDMARDs one or more times, respectively, among the registered patients in this study. All analyses were conducted using the mITT population.

### Statistical analysis

In contrast to randomized controlled trials, it is difficult to compare the efficacy in an observational study because of the treatment selection bias. Therefore, we adjusted patient characteristics between groups using propensity scores. Propensity scores were estimated using a multivariate logistic regression model predicting treatment with TCZ-SC based on the following key variables: background (age, weight, disease duration, salary, education, and occupation); concomitant use of glucocorticoids and/or methotrexate, rheumatoid factor, anti-cyclic citrullinated peptide antibody; disease activity and severity (class, stage, DAS28-ESR, CDAI, and SDAI); and questionnaires (percentage of OWI, absenteeism, presenteeism, activity impairment, EQ-5D, HAQ-DI, and K6).

The mean change from baseline and differences between treatment groups were estimated by linear regression with a robust variance estimator adjusted by the inverse probability of treatment weighting (IPTW) method. The last observation carried forward (LOCF) method was used for missing data. The primary adjustment method for confounding was changed from propensity score matching to IPTW by the research steering committee based only on baseline information, excluding post-treatment measurement values, as prespecified in the protocol.

Sensitivity analysis for estimating propensity scores confirmed the robustness of the present analyses by the model selection method (backward selection) using the clinically significant factors and selected variables. Additionally, we performed the Wilcoxon rank sum test after propensity score matching, a stratified analysis with five strata based on propensity score and regression analyses adjusting for clinically significant factors. Data insufficiently adjusted by IPTW (i.e., methotrexate yes/no) were separately and additionally adjusted by using sensitivity analysis to avoid any effects on the primary statistics.

An exploratory, linear regression analysis was conducted to investigate background factors possibly related to activity impairment and OWI improvement in PWs. The absolute standard partial regression coefficient for each baseline factor was calculated to assess the treatment response to TCZ-SC and csDMARDs. All statistical analyses were performed using SAS 9.3 version (SAS Institute Inc., Cary, NC, USA).

## Results

### Patients

**Fig. 1:**
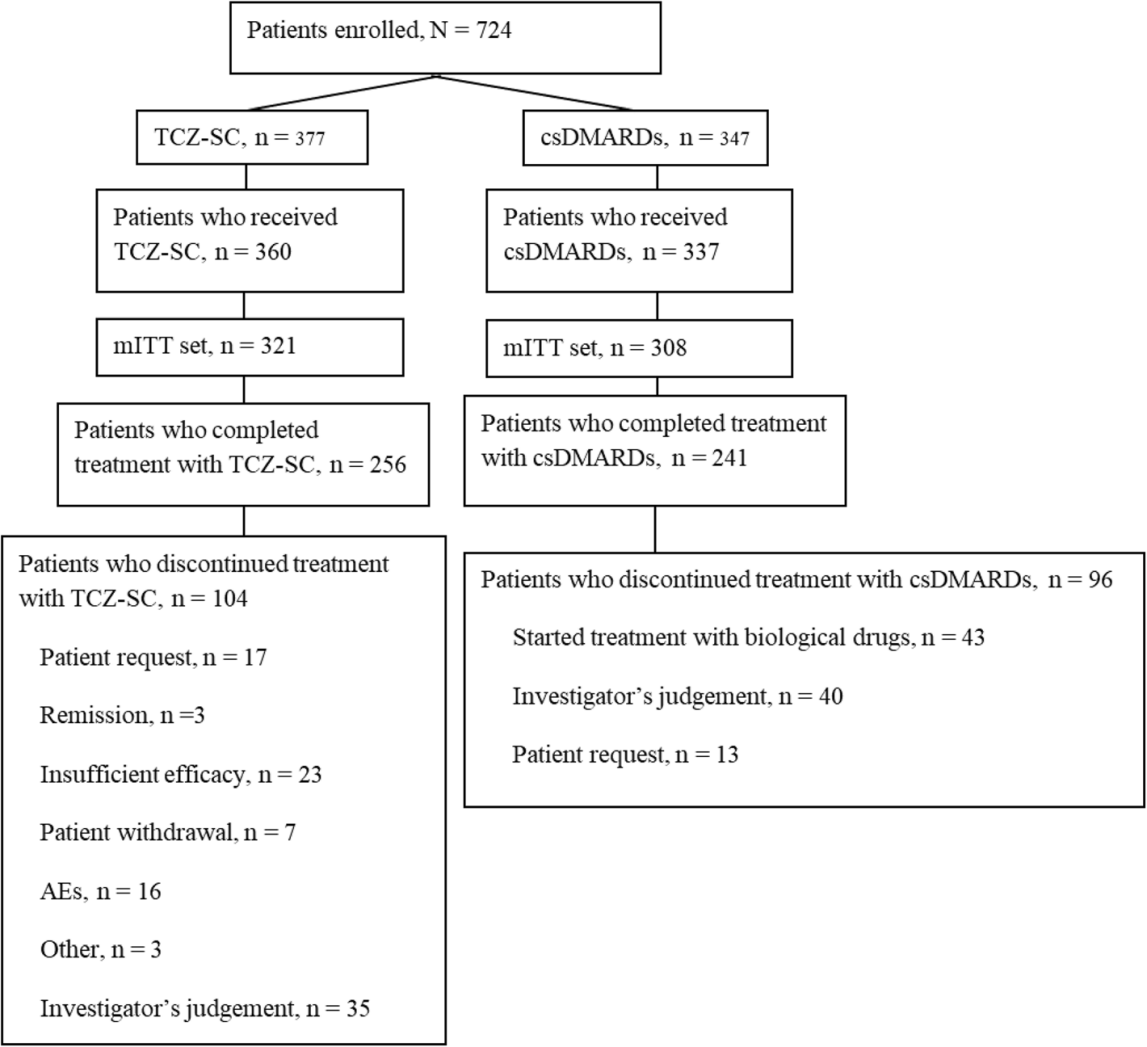
Patient disposition. AEs adverse events, csDMARDs conventional synthetic disease-modifying antirheumatic drugs, mITT modified intention-to-treat, TCZ-SC tocilizumab by subcutaneous injection

A total of 377 and 347 patients were enrolled in the TCZ-SC and csDMARDs-alone groups, respectively (Fig. 1). At 52 weeks, 256 and 241 patients, respectively, remained under study treatment in the TCZ-SC and csDMARDs-alone groups.

The main reasons for discontinuation in the TCZ-SC group were investigator’s decision (9.3%), insufficient efficacy (6.1%), patient request (4.5%), AEs (4.2%), and patient withdrawal (1.9%). In the csDMARDs-alone group, most patients discontinued because they began treatment with a biological drug (12.4%), followed by investigator decision (11.5%), and patient request (3.7%).

At baseline (unadjusted data) in the mITT population, over 75% of patients among PWs and HWs in the TCZ-SC and csDMARDs-alone groups were women (PWs 75.4% and 78.1%, HWs 88.3% and 93.9%, respectively), had a mean (± standard deviation (SD)) age of over 51 years (PWs 51.5 ± 12.1 and 55.0 ± 11.5 years, HWs 64.5 ± 12.6 and 65.5 ± 12.0 years, respectively), and a mean (± SD) disease duration of over 4 years (PWs 5.77 ± 8.23 and 4.36 ± 5.83 years, HWs 8.09 ±10.58 and 5.99 ± 7.76 years, respectively). Regarding the Steinbrocker Stage and Class and DAS28-ESR score, disease activity was higher in both PWs and HWs in the TCZ-SC group compared with PWs and HWs in the csDMARDs-alone group. The OWI of the PWs also indicated a higher impairment in the TCZ-SC group at baseline compared with the csDMARDs-alone group (Table 1). Additional file 1 shows the baseline and clinical characteristics of the mITT population after adjustment using IPTW. Most characteristics were sufficiently adjusted for by using IPTW since the absolute value of the standardized difference was lower than 0.1. In the TCZ-SC and csDMARDs-alone groups, 74.5% and 72.1% of PWs and 94.6% and 77.0% of HWs, respectively, were women. In the TCZ-SC and csDMARDs-alone groups, the mean (± SD) age of PWs was 52.2 ± 12.1 and 53.0 ± 10.9 years, respectively, and that of HWs was 64.6 ± 11.8 and 64.8 ± 11.5 years, respectively. Results for mean disease duration were also similar after adjusting for these variables (PWs 5.27 ± 7.18 and 5.28 ± 7.08 years, HWs 6.57 ± 9.87 and 6.44 ± 8.10 years). Similar results were obtained for the overall population when comparing the adjusted and unadjusted results of TCZ-SC and csDMARDs-alone groups (Additional file 2).

**Table 1:** Baseline characteristics of paid workers and house workers (unadjusted data) (modified intention-to-treat set)

	Paid worker			House worker		
	TCZ-SC group (*n* = 167)	csDMARDs-alone group (*n* = 160)	Standardized difference csDMARDs vs TCZ-SC	TCZ-SC group (*n* = 154)	csDMARDs-alone group (*n* = 148)	Standardized difference csDMARDs vs TCZ-SC
Sex, female, *n* (%)	126 (75.4)	125 (78.1)	0.063	136 (88.3)	139 (93.9)	0.198
Age (years), mean (SD)	51.5 (12.1)	55.0 (11.5)	0.299	64.5 (12.6)	65.5 (12.0)	0.085
Weight (kg), mean (SD)	57.01 (11.65)	56.02 (11.35)	−0.086	52.43 (9.22)	51.87 (10.16)	−0.058
Disease duration (years), mean (SD)	5.77 (8.23)	4.36 (5.83)	−0.197	8.09 (10.58)	5.99 (7.76)	−0.226
Income^a^, *n* (%)						
< 1,000,000 yen	5 (3.0)	8 (5.0)	0.103	15 (9.7)	13 (8.8)	−0.033
1,000,000–< 2,000,000 yen	12 (7.2)	17 (10.6)	0.121	24 (15.6)	20 (13.5)	−0.059
2,000,000–< 3,000,000 yen	18 (10.8)	23 (14.4)	0.109	35 (22.7)	24 (16.2)	−0.165
3,000,000–< 5,000,000 yen	50 (29.9)	49 (30.6)	0.015	49 (31.8)	40 (27.0)	−0.105
5,000,000–< 7,000,000 yen	37 (22.2)	25 (15.6)	−0.167	17 (11.0)	19 (12.8)	0.056
≥ 7,000,000 yen	42 (25.1)	36 (22.5)	−0.062	14 (9.1)	24 (16.2)	0.216
Unknown	3 (1.8)	2 (1.3)	−0.045	0 (0.0)	8 (5.4)	0.338
Job, *n* (%)						
Full-time/unknown	81 (48.5)	72 (45.0)	−0.070	–	–	–
Part-time	51 (30.5)	58 (36.3)	0.121	–	–	–
Private business	35 (21.0)	30 (18.8)	−0.055	–	–	–
Housework	–	–	–	154 (100.0)	148 (100.0)	–
Methotrexate, *n* (%)	130 (77.8)	153 (95.6)	0.543	98 (63.6)	133 (89.9)	0.653
Steinbrocker stage, *n* (%)						
Stage I	69 (41.3)	71 (44.4)	0.062	33 (21.4)	55 (37.2)	0.351
Stage II	54 (32.3)	60 (37.5)	0.108	66 (42.9)	46 (31.1)	−0.246
Stage III	22 (13.2)	17 (10.6)	−0.079	30 (19.5)	21 (14.2)	−0.142
Stage IV	22 (13.2)	12 (7.5)	−0.187	25 (16.2)	26 (17.6)	0.036
Steinbrocker class, *n* (%)						
Class 1	43 (25.7)	59 (36.9)	0.242	24 (15.6)	40 (27.0)	0.282
Class 2	114 (68.3)	95 (59.4)	−0.186	107 (69.5)	97 (65.5)	−0.084
Class 3/4	10 (6.0)	6 (3.8)	−0.104	23 (14.9)	11 (7.4)	−0.240
DAS28-ESR, mean (SD)	5.110 (1.261)	4.527 (0.991)	−0.514	5.546 (1.183)	4.882 (1.008)	−0.605
CDAI, mean (SD)	23.995 (11.588)	17.823 (8.713)	−0.602	26.773 (13.514)	19.053 (10.120)	−0.647
SDAI, mean (SD)	25.902 (2.852)	19.086 (9284)	−0.608	31.075 (26.411)	20.868 (10.805)	−0.506
Rheumatoid factor, *n* (%)						
Positive	112 (67.1)	100 (62.5)	−0.020	99 (64.3)	95 (64.2)	− 0.150
Negative	32 (19.2)	30 (18.8)	0.020	24 (15.6)	33 (22.3)	0.150
ACPA, *n* (%)						
Positive	100 (59.9)	78 (48.8)	−0.104	90 (64.3)	95 (64.2)	−0.123
Negative	26 (15.6)	26 (16.3)	0.104	18 (11.7)	20 (13.5)	0.123
WPAI						
Absenteeism = 0, *n* (%)	117 (70.1)	118 (73.8)	0.082	–	–	–
Absenteeism > 0, *n* (%)	44 (26.3)	37 (23.1)	−0.075	–	–	–
Presenteeism (%), mean (SD)	45.9 (32.2)	34.8 (26.9)	−0.373	–	–	–
OWI (%), mean (SD)	48.7 (32.9)	37.0 (29.2)	−0.346	–	–	–
AI (%), mean (SD)	53.6 (31.6)	40.8 (26.7)	−0.440	58.8 (27.5)	45.2 (27.9)	−0.492
WFun, mean (SD)	16.4 (8.7)	14.0 (7.5)	−0.290	–	–	–
EQ-5D, mean (SD)	0.596 (0.140)	0.656 (0.137)	0.434	0.564 (0.152)	0.654 (0.163)	0.569
HAQ-DI, mean (SD)	0.917 (0.709)	0.643 (0.585)	−0.421	1.255 (0.759)	0.959 (0.647)	−0.419

^a^100 yen = 0.9 US$

*ACPA* antibodies to citrullinated peptide antigens, *CDAI* clinical disease activity index, *csDMARD* conventional synthetic disease-modifying antirheumatic drug, *DAS28-ESR* disease activity score in 28 joints using the erythrocyte sedimentation rate, *EQ-5D* EuroQol 5 dimension, *HAQ-DI* health assessment questionnaire disability index, *SD* standard deviation, *SDAI* simplified disease activity index, *TCZ-SC* tocilizumab by subcutaneous injection, *WFun* work functioning impairment scale, *WPAI* work productivity and activity impairment questionnaire

### Efficacy

#### Primary endpoint

Table 2 summarizes the results related to the mean change in the percentage of OWI using WPAI at week 52 and adjusted using IPTW. The weighted change in OWI from baseline for PWs was −18.9% in the TCZ-SC group and −19.0% in the csDMARDs-alone group at week 52, without a significant difference between groups (adjusted treatment difference 0.1%, 95% confidence interval (CI) −6.3% to 6.5%; *P* = 0.978).

**Table 2:** Adjusted mean change in WPAI, Work Functioning Impairment Scale, DAS28-ESR, and QOL measures at week 52

	PWs or HWs	TCZ-SC group	csDMARDs-alone group	Difference between TCZ-SC group − csDMARDs-alone group (95% confidence interval)	*P* value (TCZ-SC group vs csDMARDs-alone group)
WPAI					
OWI (%)	PWs	−18.9	−19.0	0.1 (−6.3, 6.5)	0.978
Presenteeism (%)	PWs	−17.7	−17.2	−0.5 (−6.7, 5.6)	0.868
Absenteeism (%)	PWs	−7.1	−6.0	−1.1 (−4.8, 2.7)	0.580
Activity impairment (%)	Overall	−22.3	−15.8	−6.4 (−10.7, −2.2)	0.003
	PWs	−22.5	−19.4	−3.1 (−8.8, 2.7)	0.293
	HWs	−24.0	−14.5	−9.5 (−16.0, −2.9)	0.005
Work Functioning Impairment Scale					
WFun	PWs	−3.2	−3.2	0.0 (−1.3, 1.3)	0.983
Disease activity					
DAS28-ESR	Overall	−2.732	−1.388	−1.344 (−1.601, −1.087)	< 0.001
	PWs	−2.576	−1.577	−0.999 (−1.386, −0.612)	< 0.001
	HWs	−2.953	−1.279	−1.674 (−2.050, −1.298)	< 0.001
CDAI	Overall	−13.932	−10.340	−3.591 (−5.440, −1.742)	< 0.001
	PWs	−12.951	−11.679	−1.272 (−3.484, 0.939)	0.259
	HWs	−15.399	−9.427	−5.972 (−8.964, −2.980)	< 0.001
SDAI	Overall	−16.014	−11.521	−4.494 (−6.528, −2.459)	< 0.001
	PWs	−14.117	−12.338	−1.778 (−4.163, 0.607)	0.143
	HWs	−18.391	−11.388	−7.003 (−10.507, −3.499)	< 0.001
QOL					
FAI	HWs	1.8	0.7	1.0 (0.0, 2.1)	0.054
EQ-5D	Overall	0.147	0.092	0.055 (0.023, 0.086)	< 0.001
	PWs	0.154	0.123	0.031 (−0.015, 0.077)	0.182
	HWs	0.162	0.075	0.087 (0.036, 0.137)	< 0.001
HAQ-DI	Overall	−0.355	−0.238	−0.117 (−0.207, −0.027)	0.011
	PWs	−0.349	−0.286	−0.063 (−0.176, 0.050)	0.274
	HWs	−0.381	−0.226	−0.155 (−0.305, −0.005)	0.042
K6	Overall	−2.2	−1.1	−1.2 (−1.8, −0.6)	< 0.001
	PWs	−1.9	−1.2	−0.6 (− 1.3, 0.0)	0.065
	HWs	−3.0	−1.1	−1.8 (−2.9, −0.8)	< 0.001

Adjusted-weight analysis by inverse probability treatment weighting method

Last observation carried forward method was applied for missing data due to patient discontinuation

*CDAI* clinical disease activity index, *csDMARD* conventional synthetic disease-modifying antirheumatic drug, *DAS28-ESR* disease activity score in 28 joints using the erythrocyte sedimentation rate, *EQ-5D* EuroQol 5 dimension, *FAI* Frenchay activities index, *HAQ-DI* health assessment questionnaire disability index, *HW* house worker, *K6* 6-item Kessler psychological distress scale, *OWI* overall work impairment, *PW* paid worker, *QOL* quality of life, *SDAI* simplified disease activity index, *TCZ-SC* tocilizumab by subcutaneous injection, *WFun* work functioning impairment scale, *WPAI* work productivity and activity impairment questionnaire

#### Secondary endpoints for efficacy and QOL

After adjustment using IPTW, differences among PWs between the TCZ-SC and csDMARDs-alone groups in the percentage of presenteeism (−0.5%, 95% CI −6.7% to 5.6%) and absenteeism (−1.1%, 95% CI −4.8% to 2.7%) at week 52 were not significantly different (*P* = 0.868 and 0.580, respectively). The changes in WPAI activity impairment in the overall group (between-group difference −6.4%, 95% CI −10.7% to −2.2%) and in HWs (−9.0%, 95% CI −16.0% to −2.9%) were significantly better in the TCZ-SC group compared with the csDMARDs-alone group at week 52 (*P* = 0.003 and 0.005, respectively).

**Fig. 2:**
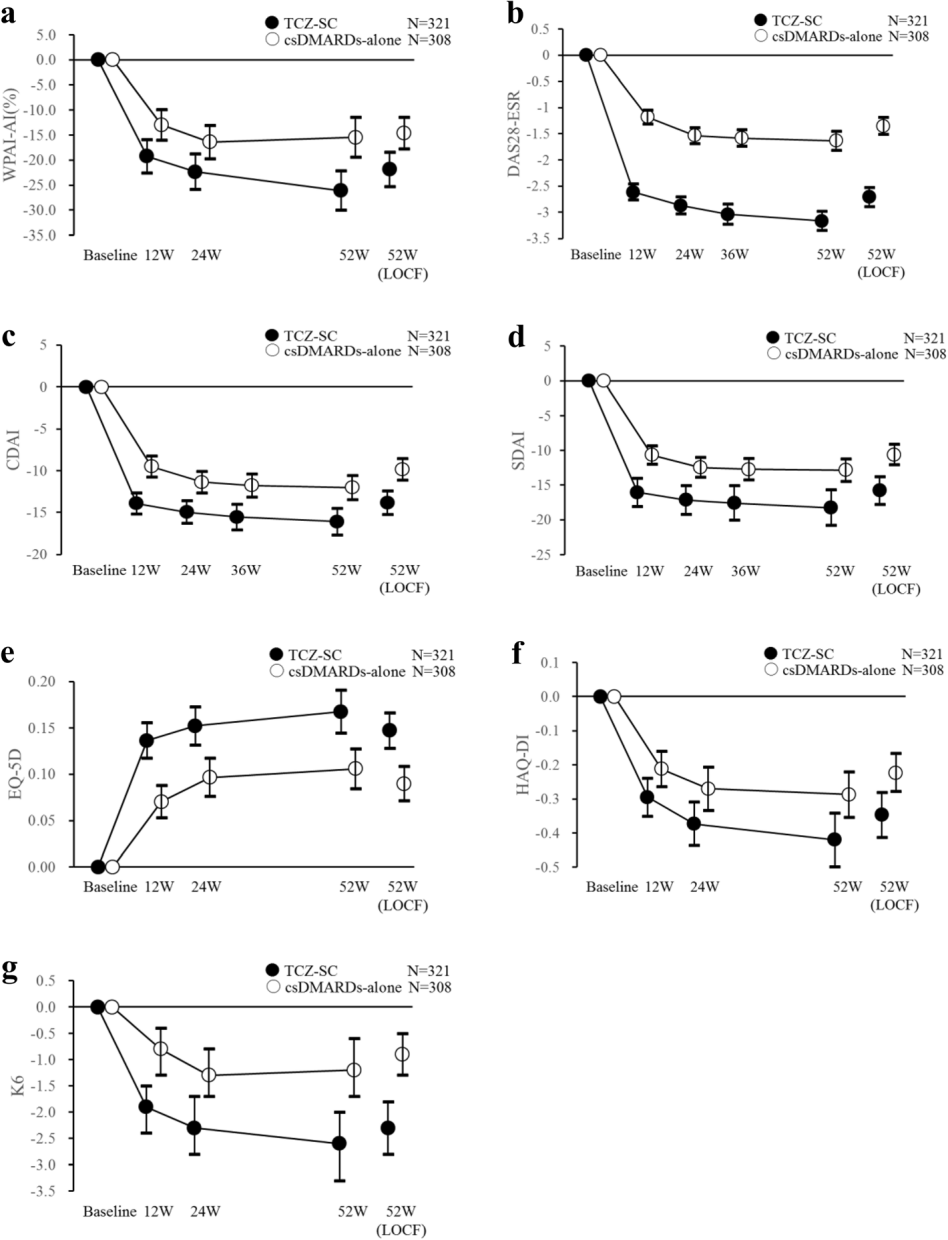
Mean change in WPAI-AI, DAS28-ESR, CDAI, SDAI, EQ-5D, HAQ-DI, and K6 over time. Mean change from baseline and 95% confidence interval in (**a**) WPAI-AI, (**b**) DAS28-ESR, (**c**) CDAI, (**d**) SDAI, (**e**) EQ-5D, (**f**) HAQ-DI, and (**g**) K6 over time (overall population) adjusted using the inverse probability of treatment weighting (IPTW) method. AI activity impairment, CDAI clinical disease activity index, csDMARDs conventional synthetic disease-modifying antirheumatic drugs, DAS28-ESR disease activity score in 28 joints using the erythrocyte sedimentation rate, EQ-5D EuroQol 5 dimension, HAQ-DI health assessment questionnaire, K6 6-item Kessler psychological distress scale, LOCF last observation carried forward, SDAI simplified disease activity index, TCZ-SC tocilizumab by subcutaneous injection, W weeks, WPAI Work Productivity and Activity Impairment

The weighted changes by IPTW method over time in WPAI, DAS28-ESR, CDAI, and SDAI in the overall population are shown in Fig. 2a–d. For these secondary endpoints, significant differences were observed in all assessments in the overall population. Improvements were observed for WPAI and disease activity (DAS28-ESR, CDAI, and SDAI), indicating improvements in treatment efficacy from baseline to 12 weeks.

Regarding the changes in disease activity according to DAS28-ESR at week 52 (Table 2), improvements in disease activity in the overall group (between-group difference −1.344, 95% CI −1.601 to −1.087; *P* <  0.001), in PWs (−0.999, 95% CI −1.386 to −0.612; *P* <  0.001), and HWs (− 1.674, 95% CI −2.050 to −1.298; *P* <  0.001) were significantly greater in the TCZ-SC group compared with the csDMARDs-alone group. Changes in disease activity according to CDAI and SDAI were only significantly different for the overall population and HWs (*P* <  0.001 for all).

**Fig. 3:**
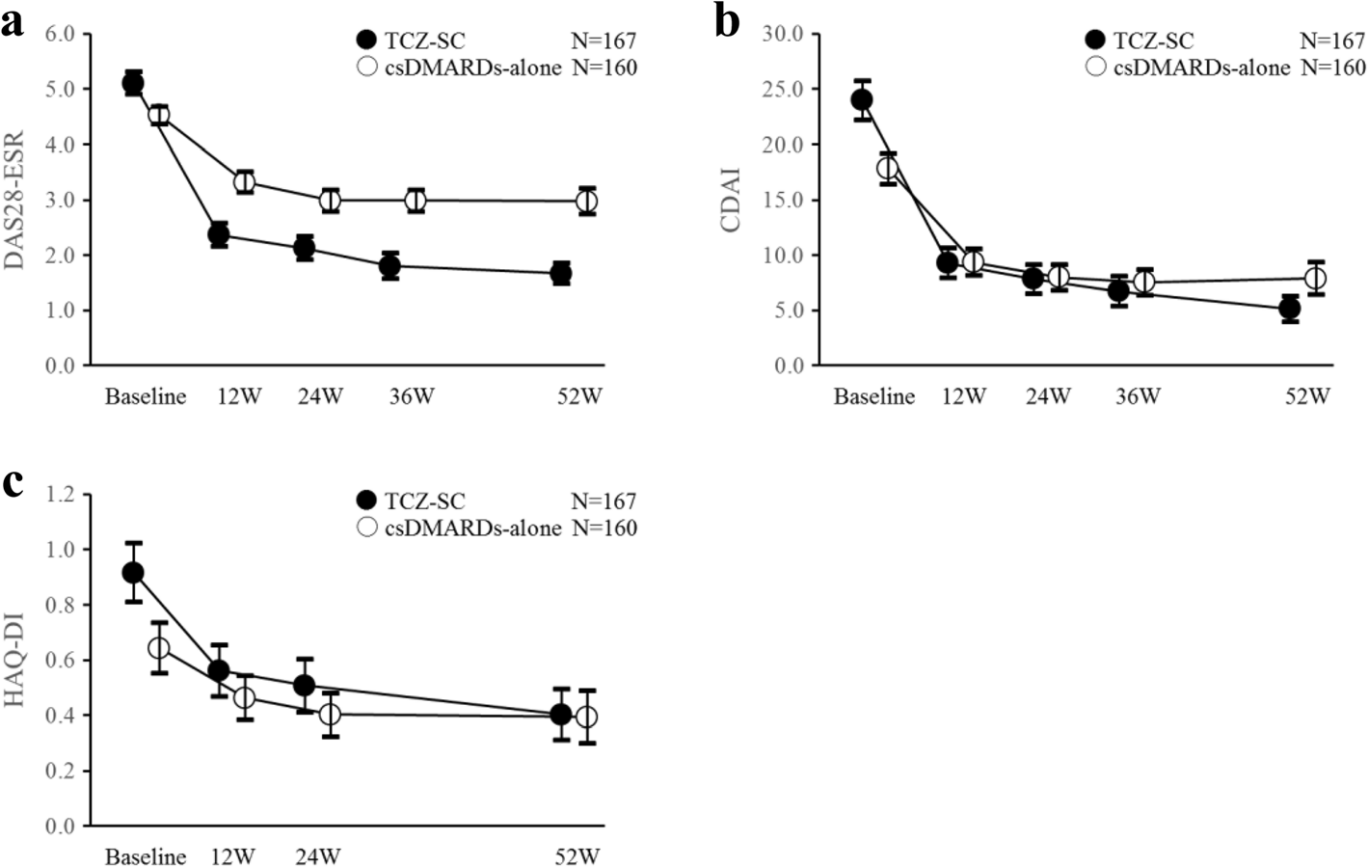
Mean change (unadjusted) in DAS28-ESR, CDAI, and HAQ-DI among paid workers. Unadjusted mean change from baseline and 95% confidence interval in (**a**) DAS28-ESR, (**b**) CDAI, and (**c**) HAQ-DI. CDAI clinical disease activity index, csDMARDs conventional synthetic disease-modifying antirheumatic drugs, DAS28-ESR disease activity score in 28 joints using the erythrocyte sedimentation rate, HAQ-DI Health Assessment Questionnaire Disability Index, TCZ-SC tocilizumab by subcutaneous injection, W weeks

The unadjusted changes in DAS28-ESR and CDAI in PWs over time indicated that disease activity decreased in both treatment groups (Fig. 3a, b). Regarding the changes in QOL measures at week 52 (Table 2), TCZ-SC-treated HWs showed significant improvement in overall QOL, as well as in FAI, EQ-5D, HAQ-DI, and K6, at week 52. PWs did not show any between-group differences for these QOL measures. There were no significant between-group differences in the changes in WFun at week 52 (0.0, 95% CI −1.3 to 1.3; *P* = 0.983).

Regarding the remission rates at week 52 (Table 3), after adjustment using IPTW and according to DAS28-ESR, significantly more patients in the overall population (67.9%), PWs (66.3%), and HWs (70.3%) treated with TCZ-SC achieved remission at week 52 (*P* <  0.0001 for all), compared with those receiving csDMARDs alone. According to CDAI and SDAI, significantly more patients in the overall population and HWs treated with TCZ-SC achieved remission at week 52 (*P* <  0.0001), compared with those receiving csDMARDs alone.

**Table 3:** Remission rate in each group by DAS28-ESR, CDAI, and SDAI

Remission rate	PWs or HWs	TCZ-SC group, *n* (%)	csDMARDs-alone group, *n* (%)	Odds ratio for TCZ-SC group/csDMARDs-alone group (95% confidence interval)	*P* value (TCZ-SC group vs csDMARDs-alone group)
DAS28-ESR	Overall	205.0 (67.9)	64.6 (21.7)	6.778 (4.508, 10.189)	< 0.0001
	PWs	104.1 (66.3)	39.7 (25.5)	5.689 (3.146, 10.287)	< 0.0001
	HWs	102.0 (70.3)	22.8 (16.1)	9.153 (4.814, 17.403)	< 0.0001
CDAI	Overall	125.5 (39.2)	58.5 (19.2)	2.549 (1.631, 3.982)	< 0.0001
	PWs	62.4 (37.3)	43.0 (27.1)	1.564 (0.860, 2.845)	0.1427
	HWs	70.2 (45.9)	16.1 (11.1)	5.635 (2.757, 11.516)	< 0.0001
SDAI	Overall	132.1 (41.8)	57.4 (19.1)	2.855 (1.828, 4.458)	< 0.0001
	PWs	65.8 (40.1)	41.6 (26.5)	1.811 (0.991, 3.309)	0.0535
	HWs	73.0 (48.1)	16.1 (11.3)	6.052 (2.981, 12.288)	< 0.0001

Adjusted odds ratio by the logistic regression model using last observation carried forward; adjusted-weight analysis by inverse probability treatment weighting method

Remission was defined as DAS28-ESR < 2.6, CDAI ≤2.8, and SDAI ≤3.3

*CDAI* clinical disease activity index, *csDMARD* conventional synthetic disease-modifying antirheumatic drug, *DAS28-ESR* disease activity score in 28 joints using the erythrocyte sedimentation rate, *HW* house worker, *PW* paid worker, *SDAI* simplified disease activity index, *TCZ-SC* tocilizumab by subcutaneous injection

Figure 2e–g shows the weighted mean changes in EQ-5D, HAQ-DI, and K6 over time in the overall population. There were improvements in QOL assessments in both treatment groups. Figure 3c shows the unadjusted changes of HAQ-DI in PWs over time. Body function, as measured by HAQ-DI, improved from baseline in both groups as well.

Additionally, we conducted exploratory analyses to identify factors possibly related to the differences in efficacy results of activity impairment (Table 4) and OWI (Table 5) and the treatment received. Regarding overall activity impairment outcomes, all parameters analyzed were significantly related to TCZ-SC treatment. However, HAQ-DI, CDAI, SDAI, and K6 did not show a significant relationship with csDMARDs alone treatment. Regarding overall work impairment outcomes, all parameters analyzed, except for K6 and CDAI, were significantly related to TCZ-SC treatment; furthermore, all parameters analyzed, except for CDAI and SDAI, were significantly related to csDMARD treatment.

**Table 4:** Exploratory analysis of relationships between overall activity impairment outcomes and type of drug received

Activity impairment measures	TCZ-SC group				csDMARDs-alone group			
	*n*	Standardized regression coefficient	95% CI	*P* value	*n*	Standardized regression coefficient	95% CI	*P* value
Presenteeism (%)	143	−0.4861	−0.6470, −0.3252	< 0.0001	148	−0.4328	−0.5887, −0.2769	< 0.0001
Overall work impairment (%)	143	−0.4819	−0.6437, −0.3202	< 0.0001	148	−0.3913	−0.5491, −0.2336	< 0.0001
Activity impairment (%)	143	−0.6345	−0.7747, −0.4943	< 0.0001	148	−0.6486	−0.7876, −0.5096	< 0.0001
HAQ-DI	143	−0.4828	−0.6415, −0.3241	< 0.0001	148	−0.1584	−0.3360, 0.0201	0.0815
EQ-5D	143	0.3635	0.1960, 0.5311	< 0.0001	148	0.3181	0.1446, 0.4916	0.0004
DAS28-ESR	143	−0.3305	−0.4950, −0.1661	0.0001	148	−0.1802	−0.3533, −0.0072	0.0414
CDAI	143	−0.1950	−0.3674, −0.0226	0.0270	148	−0.1127	−0.2819, 0.0564	0.1897
SDAI	143	−0.2289	−0.4003, −0.0576	0.0092	148	−0.1113	−0.2810, 0.0585	0.1971
WFun	143	−0.3549	−0.5266, −0.1832	< 0.0001	148	−0.1680	−0.3353, −0.0007	0.0491
K6	143	−0.1895	−0.3661, −0.0130	0.0355	148	−0.1497	−0.3216, 0.0222	0.0873

*CDAI* clinical disease activity index, *CI* confidence interval, *csDMARD* conventional synthetic disease-modifying antirheumatic drug, *DAS28-ESR* disease activity score in 28 joints using the erythrocyte sedimentation rate, *EQ-5D* EuroQol 5 dimension, *HAQ-DI* health assessment questionnaire disability index, *K6* 6-item Kessler psychological distress scale, *SDAI* simplified disease activity index, *TCZ-SC* tocilizumab by subcutaneous injection, *WFun* work functioning impairment scale

**Table 5:** Exploratory analysis of the relationships between overall work impairment outcomes and type of drug received

	TCZ-SC group				csDMARDs-alone group			
Work impairment measures	*n*	Standardized regression coefficient	95% CI	*P* value	*n*	Standardized regression coefficient	95% CI	*P* value
Presenteeism (%)	143	−0.5751	−0.7214, −0.4289	< 0.0001	147	−0.6999	−0.8240, −0.5758	< 0.0001
Overall work impairment (%)	143	−0.5950	−0.7393, −0.4507	< 0.0001	147	−0.6845	−0.8094, −0.5595	< 0.0001
Activity impairment (%)	143	−0.4754	−0.6287, −0.3221	< 0.0001	147	−0.5008	−0.6562, −0.3453	< 0.0001
HAQ-DI	143	−0.4107	−0.5707, −0.2508	< 0.0001	147	−0.1920	−0.3696, −0.0145	0.0343
EQ-5D	143	0.2840	0.1164, 0.4516	0.0011	147	0.2872	0.1126, 0.4618	0.0014
DAS28-ESR	143	−0.2961	−0.4581, −0.1342	0.0004	147	−0.2434	−0.4140, −0.0729	0.0055
CDAI	143	−0.1629	−0.3320, 0.0061	0.0587	147	−0.1608	−0.3287, 0.0070	0.0602
SDAI	143	−0.2030	−0.3709, −0.0351	0.0182	147	−0.1639	−0.3321, 0.0043	0.0560
WFun	143	−0.3748	−0.5403, −0.2092	< 0.0001	147	−0.1875	−0.3544, −0.0206	0.0280
K6	143	−0.1408	−0.3142, 0.0327	0.1108	147	−0.2002	−0.3704, − 0.0300	0.0215

Adjusted for sex, age, disease duration, job type, use of methotrexate, Steinbrocker stage, and Steinbrocker class

*CDAI* clinical disease activity index, *CI* confidence interval, *csDMARD* conventional synthetic disease-modifying antirheumatic drug, *DAS28-ESR* disease activity score in 28 joints using the erythrocyte sedimentation rate, *EQ-5D* EuroQol 5 dimension, *HAQ-DI* health assessment questionnaire disability index, *K6* 6-item Kessler psychological distress scale, *SDAI* simplified disease activity index, *TCZ-SC* tocilizumab by subcutaneous injection, *WFun* work functioning impairment scale

### Safety and adverse events

The results for AEs are summarized in Table 6. The most frequent AEs in the TCZ-SC group (*n* = 358) were nasopharyngitis (15 (4.2%)), stomatitis (11 (3.1%)), liver dysfunction (8 (2.2%)), and leukopenia (11 (3.1%)). In the csDMARDs group (*n* = 336), the most frequent AEs were nasopharyngitis (13 (3.9%)) and liver dysfunction (11 (3.3%)).

**Table 6:** Summary of adverse events in the safety analysis set

	TCZ-SC group, *n* (%)	csDMARDs-alone group, *n* (%)
*n*	358	336
AEs	127 (35.5)	99 (29.5)
Drug-related AEs	85 (23.7)	–
Serious AEs	32 (8.9)	11 (3.3)
Serious drug-related AEs	21 (5.9)	–
AEs leading to discontinuation of study treatment	33 (9.2)	28 (8.3)
Drug-related AEs leading to discontinuation of study treatment	29 (8.1)	–

AE adverse event, *csDMARD* conventional synthetic disease-modifying antirheumatic drug, *TCZ-SC* tocilizumab by subcutaneous injection

## Discussion

The present study is the first to assess the effect of TCZ-SC and/or csDMARDs on WPAI in PWs and HWs among Japanese patients with RA. We did not identify any significant difference between PWs treated with TCZ-SC and/or csDMARDs in terms of the change in OWI according to the WPAI at 52 weeks. However, we did observe an improvement in OWI from baseline in both treatment groups, meaning that RA treatment intervention was effective in decreasing disease activity, improving function, and improving overall QOL. These findings are consistent with a previous, large-scale study that evaluated the effects of adalimumab on WPAI in Japanese RA patients [5]. Previous studies of etanercept plus methotrexate in Latin America [25] and Asia [26] showed similar improvements in patient-reported outcomes, including WPAI. A previous study comparing baricitinib with placebo and adalimumab reported statistically significant improvements in absenteeism (*P* ≤ 0.05), presenteeism (*P* ≤ 0.001), and work productivity loss (*P* ≤ 0.001) with baricitinib compared with placebo; however, improvements compared with adalimumab were not statistically significant at week 52 [27].

Despite the result in OWI in the present study, improvement in the percentage of activity impairment in the overall population and among HWs was significantly better in the TCZ-SC group compared with the csDMARDs-alone group at week 52. This difference between treatment groups remained significant after adjusting for baseline characteristics using the IPTW method. This finding shows that, compared with the csDMARDs-alone treatment, TCZ-SC treatment resulted in improvement in disease activity (CDAI, SDAI, and DAS28) and significant improvement in QOL (EQ-5D, HAQ-DI, and K6). A recent, 48-week, observational study on adalimumab in Japan, focusing on work-related outcomes, showed that, compared with baseline, adalimumab treatment significantly improved measures of productivity loss due to absenteeism, presenteeism, OWI, and activity impairment in RA patients in all employment types, including PWs and HWs (*P* <  0.01) [28]. A fairly recent observational study in the US focused on work and activity impairment in employed moderate to severe RA patients and showed that etanercept led to significant reductions in overall work and activity impairment (*P* ≤ 0.0001) [1]. Furthermore, results of past studies have shown that total body inflammation and fatigue can be suppressed by inhibiting IL-6 [29]. In the present study, both clinical indexes and daily life (activity impairment) tended to improve in HWs and even in PWs. Although these changes did not reach statistical significance, these tendencies aligned with the results reported in previous studies. We consider that improvement in fatigue affected the improvement in activity impairment (daily life).

In the present study, according to DAS28-ESR, significantly more patients in the overall population, as well as PWs and HWs, treated with TCZ-SC achieved remission at week 52 (*P* <  0.0001 for all) compared with those receiving csDMARDs alone. However, by CDAI and SDAI, only those in the overall population and HWs treated with TCZ-SC achieved remission at week 52 (*P* <  0.0001). A study by Radner et al. that assessed the benefit of remission over low disease activity in RA showed that patients who achieved remission achieved better function, health-related QOL, and productivity [30].

Regarding the main differences in baseline characteristics between groups by type of work performed, HWs had greater disease severity at baseline than PWs. Similar findings were reported previously [5]. Although a tendency for improvement was observed in terms of other work productivity indices and QOL measures, no differences within the groups were observed for PWs, and a remarkable improvement in QOL measures was observed among HWs. The greater disease severity among HWs may be the reason this population experienced significantly greater improvements in overall QOL measures at week 52 compared with PWs.

An improvement in work productivity/activity impairment could be determined by differences in the treatment received as shown by the results of the presented analysis. Additionally, differences in disease severity, duration, treatment, and working conditions between PWs and HWs at baseline might have also contributed to this result. Similar conclusions were drawn in the study by Takeuchi et al. [5]. Furthermore, differences in the mechanical load on the affected joints between PWs and HWs could also be attributable to the differences in work productivity/activity impairment between PWs and HWs receiving TCZ-SC in addition to csDMARDs compared with those receiving csDMARDs alone.

Regarding the exploratory results, among PWs factors related to symptom improvement differed by study drug according to the results for HAQ-DI. These results indicated that TCZ-SC administration to patients with high HAQ-DI at baseline might result in greater improvement in work productivity and activity impairment.

The planned method for the primary analysis was changed from propensity score matching to IPTW to include all applicable patients in the analysis set because the planned sample size with a balanced number of patients was insufficient for propensity score matching. Propensity score matching allowed for easy calculation of the sample size and interpretation of the results; however, the feasibility of this method depended on whether the data of enrolled patients regarding the size of the matched sample and patient background were well balanced. For this reason, although we initially attempted to use propensity score matching, we decided to change the method after half of the patients were enrolled. In terms of bias, we consider that this change in the analysis method was acceptable because it was based on baseline data and not postbaseline data.

As part of the sensitivity analysis, we changed the adjustment method for estimating the propensity score, performed adjusted analysis with propensity score matching, used linear regression models adjusting for clinically significant factors, and performed subgroup analysis by unbalanced factors. These results were consistent with the primary results, and the robustness of the primary analysis was confirmed.

The AEs reported in the present study were in line with those previously reported for TCZ-SC in a real-world setting [31, 32]. Thus, no new safety concerns were raised, and TCZ-SC was considered a safe treatment option for Japanese RA patients.

This study had several limitations. First, the primary adjustment method for confounding was changed from propensity score matching to IPTW based on baseline data and not postbaseline data. However, we cannot deny the possibility of residual confounding effects related to the observational study design. Second, this study was conducted in a real-world clinical practice setting, and no specific criteria to initiate treatment with TCZ were applied at any of the 82 participating centers. Additionally, the dose in the TCZ-SC ± csDMARDs group was prescribed by the treating physician according to the prescribing information in the package insert; thus, we had no control over the doses prescribed. This may have affected the lower limit data of WPAI by causing a “floor effect”; in other words, patients with a low score may not have been able to show sufficient improvement. Third, we did not specifically collect information on whether a treat-to-target approach was used. However, the physicians who participated in this study were internal medicine specialists with extensive experience in the management of RA. They assessed patients at every visit (every 1–3 months). Therefore, we consider that all the patients were managed according to a treat-to-target approach. Fourth, PWs are exposed to compelling power (force) depending on the nature of the work they perform; thus, we could hardly confirm the difference between groups in terms of medical treatment. Conversely, HWs can determine their work activity level at will; thus, differences between groups in terms of the effect of medical treatment on QOL and activity level occurred easily. Fifth, WPAI is not adequate to evaluate the productivity of HWs as it was developed specifically for PWs. WPAI indexes, other than activity impairment, cannot be calculated in HWs. The other indexes are considered valid for all patients regardless of whether they are PWs or HWs. Finally, we hypothesized that the observed differences in QOL outcomes for HWs compared with PWs were related to a worst disease status in this subpopulation at baseline. Given the lack of treatment blinding, we cannot rule out other possible reasons for these outcomes such as the psychological bias and the potential emotional component related to initiating a novel treatment (e.g., biologics) that may be judged more effective than the conventional treatments. This may have affected the objectivity of the responses to the questionnaire measurements for QOL in HWs. Nevertheless, all the subjective components of CDAI and SDAI and physician's global assessment of disease activity, as well as the objective parameters, such as C-reactive peptide (CRP) and patient's global assessment of disease activity, improved with TCZ-SC in addition to csDMARDs.

## Conclusions

Despite the lack of differences in OWI between groups at week 52, the overall group (particularly HWs) receiving TCZ-SC in addition to csDMARDs showed significant improvements in activity impairment, disease activity, and QOL compared with individuals receiving csDMARDs alone. The safety of TCZ-SC was acceptable for the treatment of Japanese RA patients in a real-world clinical practice. This study may help promote the evaluation of work productivity improvements in HWs and PWs by RA treatment.

## Additional files

Additional file 1: Baseline demographic and clinical characteristics of patients in each group adjusted using inverse probability of treatment weighting in the modified intention-to-treat set (paid worker, house worker). (DOCX 22 kb)

Additional file 2: Overall baseline demographic and clinical characteristics of each group in the modified intention-to-treat set (unadjusted, adjusted). (DOCX 25 kb)

## Abbreviations

ACR: American College of Rheumatology
AE: Adverse event
CDAI: Clinical Disease Activity Index
CI: Confidence interval
csDMARD: Conventional synthetic disease-modifying antirheumatic drug
DAS28-ESR: Disease Activity Score 28-erythrocyte sedimentation rate
EQ-5D: EuroQol 5 Dimension
EULAR: European League Against Rheumatism
FAI: Frenchay Activities Index
HAQ-DI: Health Assessment Questionnaire Disability Index
HW: House worker
IL: Interleukin
IPTW: Inverse probability of treatment weighting
K6: 6-Item Kessler psychological distress scale
LOCF: Last observation carried forward
mITT: Modified intention-to-treat
OWI: Overall work impairment
PW: Paid worker
QOL: Quality of life
RA: Rheumatoid arthritis
SD: Standard deviation
SDAI: Simplified Disease Activity Index
TCZ: Tocilizumab
TCZ-SC: Tocilizumab by subcutaneous injection
VAS: Visual analog scale
WFun: Work Functioning Impairment Scale (Presenteeism Questionnaire)
WPAI: Work Productivity and Activity Impairment Questionnaire

## References

[1] Hone D, Cheng A, Watson C, Huang B, Bitman B, Huang XY (2013). Impact of etanercept on work and activity impairment in employed moderate to severe rheumatoid arthritis patients in the United States. Arthritis Care Res (Hoboken).

[2] Furuya H, Kasama T, Isozaki T, Umemura M, Otsuka K, Isojima S (2013). Effect of TNF antagonists on the productivity of daily work of patients with rheumatoid arthritis. J Multidiscip Healthc.

[3] Zhang W, Sun H, Emery P, Sato R, Singh A, Freundlich B (2012). Does achieving clinical response prevent work stoppage or work absence among employed patients with early rheumatoid arthritis?. Rheumatology (Oxford).

[4] Smolen JS, Breedveld FC, Burmester GR, Bykerk V, Dougados M, Emery P (2016). Treating rheumatoid arthritis to target: 2014 update of the recommendations of an international task force. Ann Rheum Dis.

[5] Takeuchi T, Nakajima R, Komatsu S, Yamazaki K, Nakamura T, Agata N (2017). Impact of adalimumab on work productivity and activity impairment in Japanese patients with rheumatoid arthritis: large-scale, prospective, single-cohort ANOUVEAU study. Adv Ther.

[6] Verstappen SM (2015). Rheumatoid arthritis and work: the impact of rheumatoid arthritis on absenteeism and presenteeism. Best Pract Res Clin Rheumatol.

[7] Bertin P, Fagnani F, Duburcq A, Woronoff AS, Chauvin P, Cukierman G (2016). Impact of rheumatoid arthritis on career progression, productivity, and employability: the PRET study. J Bone Spine.

[8] van Vilsteren M, Boot CR, Knol DL, van Schaardenburg D, Voskuyl AE, Steenbeek R (2015). Productivity at work and quality of life in patients with rheumatoid arthritis. BMC Musculoskelet Disord.

[9] Almoallim H, Kamil A (2013). Rheumatoid arthritis: should we shift the focus from “treat to target” to “treat to work?”. Clin Rheumatol.

[10] Rheumatoid Arthritis (RA) 2017. https://www.cdc.gov/arthritis/basics/rheumatoid-arthritis.html Accessed 2 July 2018.

[11] Tanaka E, Inoue E, Hoshi D, Shidara K, Sugimoto N, Inoue Y (2013). Assessment of work productivity and activity impairment in patients with rheumatoid arthritis based on the Institute of Rheumatology Rheumatoid Arthritis (IORRA) cohort database (FRI0124). Ann Rheum Dis.

[12] Beaton DE, Dyer S, Boonen A, Verstappen SM, Escorpizo R, Lacaille DV (2016). OMERACT filter evidence supporting the measurement of at-work productivity loss as an outcome measure in rheumatology research. J Rheumatol.

[13] Tang K, Beaton DE, Boonen A, Gignac MA, Bombardier C (2011). Measures of work disability and productivity: rheumatoid arthritis specific work productivity survey (WPS-RA), workplace activity limitations scale (WALS), work instability scale for rheumatoid arthritis (RA-WIS), work limitations questionnaire (WLQ), and work productivity and activity impairment questionnaire (WPAI). Arthritis Care Res (Hoboken)..

[14] Alghasham A, Rasheed Z (2014). Therapeutic targets for rheumatoid arthritis: progress and promises. Autoimmunity.

[15] Tanaka T, Narazaki M, Ogata A, Kishimoto T (2014). A new era for the treatment of inflammatory autoimmune diseases by interleukin-6 blockade strategy. Semin Immunol.

[16] Tocilizumab package insert for Japan, https://chugai-pharm.jp/hc/ss/pr/drug/act_via0200/pi/PDF/act_pi.pdf. Accessed 2 July 2018.

[17] Fujino Y, Uehara M, Izumi H, Nagata T, Muramatsu K, Kubo T (2015). Development and validity of a work functioning impairment scale based on the Rasch model among Japanese workers. J Occup Health.

[18] Shiratsuchi M, Saeki S, Hachisuka K (1999). Japanese version of the self-rating Frenchay activities index and its clinical application and standard values. General Rehabilitation.

[19] Tsuchiya A, Ikeda S, Ikegami N, Nishimura S, Sakai I, Fukuda T (2002). Estimating an EQ-5D population value set. The case of Japan. Health Econ.

[20] Matsuda Y, Singh G, Yamanaka H, Tanaka E, Urano W, Taniguchi A (2003). Validation of a Japanese version of the Stanford health assessment questionnaire in 3,763 patients with rheumatoid arthritis. Arthritis Rheum.

[21] Fries JF, Spitz P, Kraines RG, Holman HR (1980). Measurement of patient outcome in arthritis. Arthritis Rheum.

[22] Furukawa TA, Kessler R, Slade T, Andrews G (2003). The performance of the K6 and K10 screening scales for psychological distress in the Australian National Survey of mental health and well-being. Psychol Med.

[23] Smolen JS, Aletaha D, Bijlsma JW, Breedveld FC, Boumpas D, Burmester G (2010). Treating rheumatoid arthritis to target: recommendations of an international task force. Ann Rheum Dis.

[24] Kavanaugh A, Smolen JS, Emery P, Purcaru O, Keystone E, Richard L (2009). Effect of certolizumab pegol with methotrexate on home and work place productivity and social activities in patients with active rheumatoid arthritis. Arthritis Rheum.

[25] Machado DA, Guzman RM, Xavier RM, Simon JA, Mele L, Pedersen R (2014). Open-label observation of addition of etanercept versus a conventional disease-modifying antirheumatic drug in subjects with active rheumatoid arthritis despite methotrexate therapy in the Latin American region. J Clin Rheumatol.

[26] Bae SC, Gun SC, Mok CC, Khandker R, Nab HW, Koenig AS (2013). Improved health outcomes with etanercept versus usual DMARD therapy in an Asian population with established rheumatoid arthritis. BMC Musculoskelet Disord.

[27] Keystone EC, Taylor PC, Tanaka Y, Gaich C, DeLozier AM, Dudek A (2017). Patient-reported outcomes from a phase 3 study of baricitinib versus placebo or adalimumab in rheumatoid arthritis: secondary analyses from the RA-BEAM study. Ann Rheum Dis.

[28] Tanaka Y, Yamazaki K, Nakajima R, Komatsu S, Igarashi A, Tango T, et al. Economic impact of adalimumab treatment in Japanese patients with rheumatoid arthritis from the adalimumab non-interventional trial for up-verified effects and utility (ANOUVEAU) study. Mod Rheumatol. 2018;28(1):39–47. 10.1080/14397595.2017.1341459 28704126

[29] Choy EHS, Calabrese LH. Neuroendocrine and neurophysiological effects of interleukin 6 in rheumatoid arthritis. Rheumatology (Oxford). 10.1093/rheumatology/kex391. 10.1093/rheumatology/kex391 PMC6199533 29186541

[30] Radner H, Smolen JS, Aletaha D (2014). Remission in rheumatoid arthritis: benefit over low disease activity in patient-reported outcomes and costs. Arthritis Res Ther.

[31] Koike T, Harigai M, Inokuma S, Ishiguro N, Ryu J, Takeuchi T (2014). Effectiveness and safety of tocilizumab: postmarketing surveillance of 7901 patients with rheumatoid arthritis in Japan. J Rheumatol.

[32] Yamamoto K, Goto H, Hirao K, Nakajima A, Origasa H, Tanaka K (2015). Longterm safety of tocilizumab: results from 3 years of follow-up postmarketing surveillance of 5573 patients with rheumatoid arthritis in Japan. J Rheumatol.

