# Supplementary material for: Effect of subcutaneous tocilizumab treatment on work/housework status in biologic-naïve rheumatoid arthritis patients using inverse probability of treatment weighting: FIRST ACT-SC study
Source: Arthritis Res Ther. 2018 Jul 20;20:151. doi: 10.1186/s13075-018-1647-3 (PMC6053758; doi:10.1186/s13075-018-1647-3)
Supplement: Supplementary file 1 — Baseline demographic and clinical characteristics of patients in each group adjusted using inverse probability of treatment weighting in the modified intention-to-treat set (paid worker, house worker). (DOCX 22 kb) [file 13075_2018_1647_MOESM1_ESM.docx]

Additional File 1. Baseline demographic and clinical characteristics of patients in each group adjusted using inverse probability of treatment weighting in the modified intention-to-treat set (paid worker, house worker)

|  | **Paid worker** | | | **House worker** | | |
| --- | --- | --- | --- | --- | --- | --- |
|  | **TCZ-SC group**  **(N = 167)** | **csDMARDs-alone group**  **(N = 160)** | **Standardized difference**  **csDMARDs**  **vs TCZ-SC** | **TCZ-SC group**  **(N = 154)** | **csDMARDs-alone group**  **(N = 148)** | **Standardized difference**  **csDMARDs**  **vs TCZ-SC** |
| Sex, female, n (%) | 124.3 (74.5) | 115.3 (71.2) | 0.004 | 145.7 (94.6) | 114.0 (77.0) | −0.152 |
| Age (years), mean (SD) | 52.2 (12.1) | 53.0 (10.9) | 0.073 | 64.6 (11.8) | 64.8 (11.5) | 0.020 |
| Weight (kg), mean (SD) | 56.44 (10.95) | 55.63 (11.01) | −0.074 | 53.06 (9.46) | 54.75 (12.97) | 0.150 |
| Disease duration (years), mean (SD) | 5.27 (7.18) | 5.28 (7.08) | 0.001 | 6.57 (9.87) | 6.44 (8.10) | −0.014 |
| Income^a^, n (%) |  |  |  |  |  |  |
| <1,000,000 yen | 9.2 (5.5) | 5.7 (3.6) | −0.091 | 12.7 (8.2) | 18.0 (12.2) | 0.185 |
| 1,000,000–<2,000,000 yen | 11.6 (6.9) | 12.6 (7.9) | 0.048 | 20.5 (13.3) | 16.2 (10.9) | −0.018 |
| 2,000,000–<3,000,000 yen | 14.4 (8.6) | 15.6 (9.8) | 0.053 | 30.6 (19.9) | 25.9 (17.5) | 0.012 |
| 3,000,000–<5,000,000 yen | 49.0 (29.3) | 44.9 (28.0) | −0.007 | 46.4 (30.2) | 40.0 (27.0) | 0.027 |
| 5,000,000–<7,000,000 yen | 31.0 (18.6) | 24.6 (15.3) | −0.074 | 24.1 (15.7) | 14.4 (9.7) | −0.125 |
| ≥7,000,000 yen | 38.1 (22.8) | 38.3 (24.0) | 0.048 | 25.9 (16.8) | 18.0 (12.2) | −0.073 |
| Unknown | 4.5 (2.7) | 4.4 (2.7) | 0.009 | 0 (0.0) | 0 (0.0) | 0.185 |
| Job, n (%) |  |  |  |  |  |  |
| Full-time/unknown | 75.0 (44.9) | 67.1 (42.0) | −0.032 | – | – | – |
| Part-time | 50.2 (30.1) | 48.2 (30.1) | 0.025 | – | – | – |
| Private business | 32.5 (19.5) | 30.8 (19.2) | 0.011 | – | – | – |
| Housework | – | – | – | 160.3 (104.1) | 132.4 (89.5) | – |
| Methotrexate, n (%) | 135.8 (81.3) | 133.4 (83.3) | 0.166 | 123.1 (79.9) | 106.6 (72.0) | 0.091 |
| Steinbrocker Stage, n (%) |  |  |  |  |  |  |
| Stage I | 68.1 (40.8) | 66.7 (41.7) | 0.050 | 60.8 (39.5) | 42.7 (28.8) | −0.120 |
| Stage II | 54.8 (32.8) | 46.8 (29.3) | −0.056 | 53.3 (34.6) | 49.6 (33.5) | 0.088 |
| Stage III | 20.4 (12.2) | 21.1 (13.2) | 0.043 | 23.0 (14.9) | 17.1 (11.5) | −0.042 |
| Stage IV | 14.6 (8.7) | 11.5 (7.2) | −0.048 | 23.2 (15.1) | 23.1 (15.6) | 0.080 |
| Steinbrocker Class, n (%) |  |  |  |  |  |  |
| Class1 | 54.3 (32.5) | 46.2 (28.9) | −0.059 | 46.0 (29.9) | 27.6 (18.7) | −0.182 |
| Class2 | 95.3 (57.1) | 91.6 (57.3) | 0.048 | 97.8 (63.5) | 90.6 (61.2) | 0.155 |
| Class3/4 | 8.1 (4.9) | 8.2 (5.1) | 0.020 | 16.5 (10.7) | 14.2 (9.6) | 0.014 |
| DAS28-ESR, mean (SD) | 4.793 (1.189) | 4.739 (1.102) | −0.047 | 5.191 (1.140) | 5.193 (1.178) | 0.002 |
| CDAI, mean (SD) | 20.758 (10.796) | 20.281 (9.707) | −0.046 | 22.864 (12.391) | 22.590 (11.956) | −0.023 |
| SDAI, mean (SD) | 22.262 (11.909) | 21.520 (10.304) | −0.067 | 25.735 (21.174) | 25.250 (14.175) | −0.027 |
| Rheumatoid factor, n (%) |  |  |  |  |  |  |
| Positive | 105.4 (63.1) | 95.5 (59.7) | −0.033 | 100.4 (65.2) | 87.7 (59.2) | 0.078 |
| Negative | 25.7 (15.4) | 25.3 (15.8) | 0.033 | 33.1 (21.5) | 24.0 (16.2) | −0.078 |
| ACPA, n (%) |  |  |  |  |  |  |
| Positive | 84.4 (50.5) | 78.6 (49.1) | −0.017 | 74.8 (48.6) | 70.1 (47.3) | 0.033 |
| Negative | 22.4 (13.4) | 21.8 (13.6) | 0.017 | 18.1 (11.8) | 15.6 (10.5) | −0.033 |
| WPAI |  |  |  |  |  |  |
| Absenteeism=0, n (%) | 119.9 (71.8) | 106.4 (66.5) | −0.072 | – | – | – |
| Absenteeism >0, n (%) | 37.9 (22.7) | 39.7 (24.8) | 0.072 | – | – | – |
| Presenteeism (%), mean (SD) | 39.5 (31.1) | 37.7 (25.9) | −0.063 | – | – | – |
| OWI (%), mean (SD) | 41.9 (32.6) | 40.7 (28.0) | −0.039 | – | – | – |
| AI (%), mean (SD) | 46.4 (32.1) | 45.6 (26.5) | −0.025 | 51.9 (28.8) | 50.6 (27.5) | −0.049 |
| WFun, mean (SD) | 14.9 (8.1) | 14.9 (7.3) | -0.008 | – | – | – |
| EQ5D, mean (SD) | 0.629 (0.143) | 0.636 (0.134) | 0.052 | 0.605 (0.143) | 0.612 (0.154) | 0.050 |
| HAQ-DI, mean (SD) | 0.751 (0.661) | 0.716 (0.578) | −0.056 | 1.047 (0.763) | 1.017 (0.606) | −0.044 |

Abbreviations: TCZ-SC, tocilizumab subcutaneous injection; csDMARD, conventional synthetic disease-modifying antirheumatic drug; CI, confidence interval; SD, standard deviation; DAS28-ESR, disease activity score in 28 joints using the erythrocyte sedimentation rate; CDAI, clinical disease activity index; SDAI, simplified disease activity index; ACPA, antibodies to citrullinated peptide antigens; WPAI, Work Productivity and Activity Impairment Questionnaire; OWI, overall work impairment; EQ-5D, EuroQol 5 dimension; HAQ-DI, Health Assessment Questionnaire Disability Index; WFun, Work Functioning Impairment scale
