# Supplementary material for: Effect of subcutaneous tocilizumab treatment on work/housework status in biologic-naïve rheumatoid arthritis patients using inverse probability of treatment weighting: FIRST ACT-SC study
Source: Arthritis Res Ther. 2018 Jul 20;20:151. doi: 10.1186/s13075-018-1647-3 (PMC6053758; doi:10.1186/s13075-018-1647-3)
Supplement: Supplementary file 2 — Overall baseline demographic and clinical characteristics of each group in the modified intention-to-treat set (unadjusted, adjusted). (DOCX 25 kb) [file 13075_2018_1647_MOESM2_ESM.docx]

Additional File 2. Overall baseline demographic and clinical characteristics of each group in the modified intention-to-treat set (unadjusted, adjusted)

|  | **Unadjusted** | | | **Adjusted** | | |
| --- | --- | --- | --- | --- | --- | --- |
|  | **TCZ-SC group**  **(N=321)** | **csDMARDs-alone group**  **(N=308)** | **Standardized difference**  **csDMARDs**  **vs TCZ-SC** | **TCZ-SC group**  **(N=321)** | **csDMARDs-alone group**  **(N=308)** | **Standardized difference**  **csDMARDs**  **vs TCZ-SC** |
| Sex, female, n (%) | 262 (81.6) | 264 (85.7) | 0.111 | 271.1 (84.5) | 241.1 (78.3) | −0.032 |
| Age (years), mean (SD) | 57.7 (14.0) | 60.1 (12.8) | 0.175 | 58.2 (13.3) | 59.2 (12.6) | 0.074 |
| Weight (kg), mean (SD) | 54.81 (10.78) | 54.02 (10.97) | −0.072 | 54.39 (10.45) | 54.79 (11.66) | 0.036 |
| Disease duration (years), mean (SD) | 6.89 (9.50) | 5.14 (6.85) | −0.212 | 6.07 (8.47) | 6.08 (7.80) | 0.001 |
| Income^a^, n (%) |  |  |  |  |  |  |
| <1,000,000 yen | 20 (6.2) | 21 (6.8) | 0.024 | 19.7 (6.1) | 19.6 (6.4) | 0.026 |
| 1,000,000–<2,000,000 yen | 36 (11.2) | 37 (12.0) | 0.025 | 32.8 (10.2) | 30.1 (9.8) | 0.006 |
| 2,000,000–<3,000,000 yen | 53 (16.5) | 47 (15.3) | −0.034 | 50.1 (15.6) | 47.9 (15.6) | 0.026 |
| 3,000,000–<5,000,000 yen | 99 (30.8) | 89 (28.9) | −0.043 | 96.9 (30.2) | 86.6 (28.1) | −0.006 |
| 5,000,000–<7,000,000 yen | 54 (16.8) | 44 (14.3) | −0.070 | 51.2 (15.9) | 42.2 (13.7) | −0.038 |
| ≥7,000,000 yen | 56 (17.4) | 60 (19.5) | 0.052 | 62.0 (19.3) | 59.2 (19.2) | 0.028 |
| Unknown | 3 (0.9) | 10 (3.2) | 0.162 | 7.5 (2.3) | 3.3 (1.1) | −0.093 |
| Job, n (%) |  |  |  |  |  |  |
| Full-time/unknown | 81 (25.2) | 72 (23.4) | −0.043 | 79.3 (24.7) | 68.1 (22.1) | −0.028 |
| Part-time | 51 (15.9) | 58 (18.8) | 0.078 | 51.6 (16.1) | 48.2 (15.7) | 0.016 |
| Private business | 35 (10.9) | 30 (9.7) | −0.038 | 37.6 (11.7) | 31.8 (10.3) | −0.023 |
| Housework | 154 (48.0) | 148 (48.1) | 0.002 | 151.8 (47.3) | 140.8 (45.7) | 0.027 |
| Methotrexate, n (%) | 228 (71.0) | 286 (92.9) | 0.592 | 261.9 (81.6) | 246.0 (79.9) | 0.090 |
| Steinbrocker Stage, n (%) |  |  |  |  |  |  |
| Stage I | 102 (31.8) | 126 (40.9) | 0.191 | 126.8 (39.5) | 111.0 (36.1) | −0.024 |
| Stage II | 120 (37.4) | 106 (34.4) | −0.062 | 112.8 (35.1) | 98.0 (31.8) | −0.028 |
| Stage III | 52 (16.2) | 38 (12.3) | −0.111 | 42.2 (13.2) | 40.1 (13.0) | 0.020 |
| Stage IV | 47 (14.6) | 38 (12.3) | −0.067 | 38.4 (12.0) | 39.8 (12.9) | 0.053 |
| Steinbrocker class, n (%) |  |  |  |  |  |  |
| Class 1 | 67 (20.9) | 99 (32.1) | 0.257 | 96.8 (30.2) | 77.4 (25.1) | −0.077 |
| Class 2 | 221 (68.8) | 192 (62.3) | −0.137 | 197.5 (61.5) | 182.7 (59.3) | 0.033 |
| Class 3/4 | 33 (10.3) | 17 (5.5) | −0.177 | 25.9 (8.1) | 28.8 (9.3) | 0.066 |
| DAS28-ESR, mean (SD) | 5.320 (1.241) | 4.696 (1.013) | −0.550 | 4.964 (1.218) | 4.933 (1.122) | −0.027 |
| CDAI, mean (SD) | 25.323 (12.603) | 18.412 (9.417) | −0.621 | 21.813 (11.559) | 21.265 (10.720) | −0.049 |
| SDAI, mean (SD) | 28.390 (20.653) | 19.935 (10.061) | −0.520 | 24.007 (16.966) | 23.142 (11.914) | −0.059 |
| Rheumatoid factor, n (%) |  |  |  |  |  |  |
| Positive | 211 (65.7) | 195 (63.3) | −0.082 | 208.8 (65.0) | 187.2 (60.8) | −0.030 |
| Negative | 56 (17.4) | 63 (20.5) | 0.082 | 56.8 (17.7) | 54.8 (17.8) | 0.030 |
| ACPA, n (%) |  |  |  |  |  |  |
| Positive | 190 (59.2) | 151 (49.0) | −0.112 | 162.0 (50.5) | 155.1 (50.3) | 0.004 |
| Negative | 44 (13.7) | 46 (14.9) | 0.112 | 41.3 (12.9) | 39.1 (12.7) | −0.004 |
| WPAI |  |  |  |  |  |  |
| Absenteeism=0, n (%) | 117 (36.4) | 118 (38.3) | 0.039 | 123.8 (38.6) | 104.9 (34.1) | −0.049 |
| Absenteeism >0, n (%) | 44 (13.7) | 37 (12.0) | −0.051 | 39.8 (12.4) | 39.3 (12.8) | 0.035 |
| Presenteeism (%), mean (SD) | 45.9 (32.2) | 34.8 (26.9) | −0.373 | 39.0 (31.5) | 37.9 (25.9) | −0.039 |
| OWI (%), mean (SD) | 48.7 (32.9) | 37.9 (29.2) | −0.346 | 41.6 (32.8) | 40.9 (28.2) | −0.021 |
| AI (%), mean (SD) | 56.1 (29.8) | 42.9 (27.3) | −0.463 | 48.8 (30.6) | 47.5 (26.9) | −0.046 |
| WFun, mean (SD) | 16.4 (8.7) | 14.0 (7.5) | −0.290 | 14.9 (8.2) | 14.9 (7.4) | −0.005 |
| EQ5D, mean (SD) | 0.581 (0.146) | 0.655 (0.150) | 0.502 | 0.616 (0.147) | 0.625 (0.145) | 0.063 |
| HAQ-DI, mean (SD) | 1.079 (0.752) | 0.796 (0.635) | −0.407 | 0.919 (0.728) | 0.904 (0.64) | −0.021 |

^a^100 yen = 0.9 US$

Abbreviations: TCZ-SC, tocilizumab subcutaneous injection; csDMARD, conventional synthetic disease-modifying antirheumatic drug; CI, confidence interval; SD, standard deviation; DAS28-ESR, disease activity score in 28 joints using the erythrocyte sedimentation rate; CDAI, clinical disease activity index; SDAI, simplified disease activity index; ACPA, antibodies to citrullinated peptide antigens; WPAI, Work Productivity and Activity Impairment Questionnaire; EQ-5D, EuroQol 5 dimension; HAQ-DI, Health Assessment Questionnaire Disability Index; OWI, overall work impairment, WFun, Work Functioning Impairment scale
